# Mechanical properties of additively manufactured zirconia with alumina air abrasion surface treatment

**DOI:** 10.1038/s41598-023-36181-6

**Published:** 2023-06-06

**Authors:** Lee-Gang Yoo, Nan-Sim Pang, So-Hyun Kim, Bock-Young Jung

**Affiliations:** 1 Department of Advanced General Dentistry, College of Dentistry, Yonsei University, 50 Yonsei-Ro, Seodaemun-Gu, Seoul, 03722 South Korea; 2 Department of Dentistry, Inha University Hospital, Inha University School of Medicine, Incheon, Korea

**Keywords:** Dental biomaterials, Implants, Biological techniques

## Abstract

This study aimed to evaluate the mechanical properties of zirconia fabricated using additive manufacturing technology and compare them to those of zirconia fabricated using subtractive manufacturing technology. Sixty disc-shaped specimens were fabricated for the additive (n = 30) and subtractive manufacturing groups (n = 30), and each group was divided into two subgroups according to their air-abrasion surface treatment: control (n = 15) and air-abrasion groups (n = 15). Mechanical properties including the flexural strength (FS), Vickers hardness, and surface roughness were determined, and the values were analyzed by one-way ANOVA and Tukey’s post hoc test (α = 0.05). X-ray diffraction and scanning electron microscopy were used for phase analysis and surface topography evaluation, respectively. The SMA group exhibited the highest FS (1144.97 ± 168.1 MPa), followed by the SMC (944.58 ± 141.38 MPa), AMA (905.02 ± 111.38 MPa), and AMC groups (763.55 ± 68.69 MPa). The Weibull distribution showed the highest scale value (1213.55 MPa) in the SMA group, with the highest shape value in the AMA group (11.69). A monoclinic peak was not detected in both the AMC and SMC groups, but after air abrasion, the monoclinic phase content (${\mathrm{X}}_{\mathrm{m}}$) reached 9% in the AMA group, exceeding that in the SMA group (7%). The AM groups exhibited statistically lower FS values than those of the SM groups under the same surface treatment (p < 0.05). Air-abrasion surface treatment increased the monoclinic phase content and FS (p < 0.05) in both the additive and subtractive groups, while it increased the surface roughness (p < 0.05) only in the additive group and did not affect the Vickers hardness in either group. For zirconia manufactured using additive technology, the mechanical properties are comparable to those of zirconia manufactured using subtractive technology.

## Introduction

Zirconia-based ceramics, especially 3% yttria-tetragonal zirconia polycrystals (3Y-TZP), have become commonly adopted materials in prosthetic and implant dentistry owing to their outstanding biocompatibility, aesthetics, and mechanical properties^1,2^. Their mechanical superiority stems from the spontaneous phase transformations of tetragonal phases into monoclinic phases inside zirconia: the zirconia grain volume expands by 3 ~ 5%, which generates compressive stress and prevents crack propagation; consequently, zirconia exhibits high strength and toughness^3^.

Most zirconia dental prostheses are fabricated by subtractive computer-aided manufacturing (CAM) using presintered or fully sintered zirconia blocks^2,4–6^. Subtractive manufacturing (SM) is considered a reliable technique that enables fast and standardized production^6^. In addition, a recent systematic review showed that the accuracy of SM zirconia prostheses was mostly within 60 μm in regard to marginal, internal, and total gaps, which is within the clinically acceptable range (between 50 and 120 μm)^7^. SM technology using fully sintered blocks eliminates the need for subsequent sintering processes and prevents shrinkage, resulting in increased precision and accuracy; however, this technology exhibits several manufacturing limitations, including material waste, a short lifetime of tooling burs, surface microcracks due to the milling process, and space limitations imposed by the milling bar size and the axis of the computer numerical control (CNC) machine^6,8,9^. Moreover, using a presintered block is not recommended with this technology because thermal shrinkage in the subsequent sintering process may affect the marginal accuracy by approximately 20%^6,8,9^.

Additive manufacturing (AM) has been proposed as another method of processing zirconia prostheses to overcome the limitations of SM technology^2,9^. Moreover, it can be used to fabricate more accurate objects with complex geometries^2^ and avoid the amassment of tooling stresses related to milling^8^. Therefore, AM is regarded as a prospective technology for the fabrication of zirconia materials in the dental field because it enables mass customization with high efficiency, repeatability, and reproducibility^10^.

Among the vat photopolymerization methods of AM technology^11^, stereolithography apparatus (SLA) and digital light processing (DLP) technologies have been widely used for the fabrication of zirconia prostheses, using a ceramic slurry comprising photosensitive liquid resin and zirconia powder^12^. DLP uses digital light projection to induce the polymerization of entire layers^12^. The accuracy of zirconia prostheses depends on the manufacturing technique, printing parameters, sintering procedure, photopolymerizable ceramic suspension, and postprocessing process^9,13–15^.

A few studies have reported the mechanical properties of zirconia manufactured using AM technology, such as the flexural strength (FS) and surface hardness. The reported FS values were in the range required for SM dental zirconia (800–1200 MPa)^5,16,17^, and the reported shear bond strength between veneering feldspathic ceramics and AM zirconia frameworks was 19.9 ± 6.9 MPa^18^, which is within the specified range (16 to 42 MPa)^19^ for all-ceramic restorations demonstrated in a previous study, and that between resin cement and AM zirconia was 8.629 ± 0.914 MPa^20^.

The low adhesive strength between zirconia and resin cement has been studied, and various mechanical and chemical methods, such as air abrasion, silanization, hydrofluoric acid treatment, and the application of 10-methacryloyloxydecyl dihydrogen phosphate (10-MDP), have been suggested to improve the adhesive strength^20–23^. Air abrasion with aluminum oxide particles can increase the surface area for bonding by forming micron-sized irregularities and promoting the micromechanical retention of the applied resin cement^22–25^. However, the effects of air abrasion on the mechanical strength of SM zirconia remain controversial^22,24^. Various processing parameters, including the particle size, blasting time, air pressure, and aging, affect the FS and adhesive strength, and alumina particle sizes of 50–250 μm, air pressures ranging from 0.2 to 0.4 MPa, and blasting times of 10–20 s have been used for air abrasion^22–25^.

Although a number of previous studies have reported the mechanical properties of milled dental zirconia, there is a lack of information on the properties of zirconia fabricated using AM technology, which has recently been actively developed. In particular, few studies have reported the effect of air-abrasion surface treatment. Therefore, the purpose of the present study is to evaluate and compare the mechanical properties, i.e., FS, Vickers hardness, and surface roughness, of zirconia fabricated by AM and SM technologies.

The first null hypothesis was that there are no significant differences among the mechanical properties of zirconia fabricated with AM and SM technologies. The second was that air-abrasion surface treatment imposed no effect on the mechanical properties of zirconia fabricated using AM and SM technologies.

## Materials and methods

### Test specimens

Information on the material used in this study is provided in Table 1. All specimens were prepared according to the standards of ISO 6872. A total of 60 disc-shaped (14 mm diameter, 1.2 mm thickness) specimens were fabricated for the additive (AM: n = 30) and subtractive manufacturing groups (SM: n = 30), and each group was divided into two subgroups according to the air-abrasion surface treatment: control (AMC, SMC: n = 15) and air-abrasion groups (AMA, SMA: n = 15), forming 4 test groups. The sample size in this study was calculated using G*Power statistical software (Version 3.1.9.7, Dusseldorf, Germany) with a significance level of 0.05 and a power of 80.

**Table 1:** Information on material used in this study.

Characteristics	AM group (3D printing)	SM group (milling)
Manufacturing	DLP additive technology	Subtractive technology
Composition	Zirconia stabilized with 3% yttria	Zirconia stabilized with 3% yttria
Grain size	0.2 ~ 0.3 μm	0.15 ~ 0.2 μm
Manufacturer	Aon, Korea	Genoss, Korea

*AM* additive manufacturing, *SM* subtractive manufacturing, *DLP* digital light processing.

In the SM group, the specimens were fabricated from presintered 3Y-TZP (Rainbow block, Genoss, Korea) by a 5-axis milling machine (Sirona inLab MC X5, Dentsply Sirona, USA) and sintered in a furnace at 1500 °C for 90 min. In the AM group, zirconia slurry (INNI-CERA, AON, Korea) was additively manufactured in the horizontal direction by using a DLP 3D printer (ZIPRO, AON, Korea). Then, the specimens were debinded at 500 °C for 1 h and sintered at 1500 °C for 2 h. All specimens were wet polished with 30 ~ 40 μm diamond slurry and finally wet polished with 15 ~ 20 μm diamond slurry (Kemet, Kemet International Ltd., UK) with the exception of two specimens that were used to obtain clear SEM images and surface roughness data. Both sides of the specimen surface were flat and parallel within 0.05 mm and thoroughly washed so that all residual traces were removed. The air-abrasion groups were air-abraded on one side with 50 μm Al_2_O_3_ particles from a distance of 10 mm at a pressure of 0.2 MPa for 20 s. Each specimen, which was fixed by a zig, was treated by the air-abrasion unit moving from the left side to the right side, and to the left side according to the manufacturer’s instructions. The air-abrasion procedures were performed by one experienced researcher (L.G.Y.). Each specimen was evaluated to determine whether the entire area was air abraded based on the loss of gloss. Finally, the specimens were washed under running water for 30 s, ultrasonically washed in distilled water for 10 min, and air dried^18,22,26^. All specimens were visually inspected and excluded in case of macroscopic flaws and defects.

### Biaxial flexural strength test

The biaxial flexural strength test was performed according to ISO 6872 using a universal testing machine (Zwick Z010, Zwick/Roell, Germany) with a 1 mm/min crosshead speed until the specimen fractured. The specimens were placed on three stainless-steel balls with a diameter of 3.2 mm. In the air-abrasion group, the air-abraded surface was positioned toward the stainless-steel ball, and a test load was applied to the opposite surface. The load-at-fracture was recorded, and the FS can be calculated as follows:

1 $$\mathrm{S}=-0.2387\mathrm{ P}(\mathrm{X}-\mathrm{Y})/{\mathrm{d}}^{2}$$

where $\mathrm{S}$ is the FS (MPa), $\mathrm{P}$ is the fracture load (N), and $\mathrm{d}$ is the thickness of the disc specimen (mm). $\mathrm{X}$ and $\mathrm{Y}$ can be calculated as follows:

2 $$\mathrm{X}=\left(1+\mathrm{v}\right)\mathrm{ ln}{\left({\mathrm{r}}_{2}/{\mathrm{r}}_{3}\right)}^{2}+\left[1-\mathrm{v}/1-\mathrm{v}\right]{\left({\mathrm{r}}_{2}/{\mathrm{r}}_{3}\right)}^{2}$$

3 $$\mathrm{Y}=\left(1+\mathrm{v}\right) \left[1+\mathrm{ln}{\left({\mathrm{r}}_{1}/{\mathrm{r}}_{3}\right)}^{2}\right]+\left(1-\mathrm{v}\right){\left({\mathrm{r}}_{1}/{\mathrm{r}}_{3}\right)}^{2}$$

where $\mathrm{v}$ is Poisson’s ratio (0.25), ${\mathrm{r}}_{1}$ is the radius of the support circle, ${\mathrm{r}}_{2}$ is the radius of the load piston, and ${\mathrm{r}}_{3}$ is the radius of the specimen.

### Weibull distribution

The Weibull distribution, including shape and scale factors, was calculated using maximum likelihood estimation with a statistical software program (Minitab Software V.16, Minitab, USA). A material with a high shape shows a steep slope in the probability plot of the FS, indicating that a fracture may occur over small parts of the test specimen. The scale is the strength value at a probability of failure of 63.2%^27^.

### Vickers hardness test

The microhardness was determined using a digital Vickers hardness tester (MMT-X7B, Matsuzawa, Japan) by performing 3 measurements per specimen. The measurements were performed with a diamond pyramid tip with a square cross section by applying 1000 gf for 15 s.

### X-ray diffraction (XRD)

The phase transformation was assessed by X-ray diffraction (XRD) (Ultima IV, Rigaku, Japan) for 1 specimen per group. Scanning was performed with a step size of 0.02° for 0.6 s at 40 kV and 30 mA. The diffraction angle range ranged from 20° to 40°. The ratio of the monoclinic peak intensity ${\mathrm{X}}_{\mathrm{m}}$ was calculated according to the formula reported by Gravie and Nicholson^28^ as follows:

4 $${\mathrm{X}}_{\mathrm{m}}={\mathrm{I}}_{\mathrm{m}}\left(\overline{1 }11\right)+{\mathrm{I}}_{\mathrm{m}}(111)/{\mathrm{I}}_{\mathrm{m}}\left(\overline{1 }11\right)+{\mathrm{I}}_{\mathrm{m}}\left(111\right)+{\mathrm{I}}_{\mathrm{t}}(111)$$

where ${\mathrm{I}}_{\mathrm{m}}\left(\overline{1 }11\right)$ and ${\mathrm{I}}_{\mathrm{m}}(111)$ are the intensities of the monoclinic peaks at 2Ø = 28.2° and 31.4°, respectively, and ${\mathrm{I}}_{\mathrm{t}}(111)$ is the intensity of the tetragonal peak at 2Ø = 30.3°.

The volumetric percentage of the monoclinic phase content ${\mathrm{V}}_{\mathrm{m}}$ was calculated according to the formula reported by Toraya et al.^29^ as follows:

5 $${\mathrm{V}}_{\mathrm{m}}=1.311{\mathrm{X}}_{\mathrm{m}}/1+0.311{\mathrm{X}}_{\mathrm{m}}$$

### Surface roughness

The surface roughness of 2 specimens from each group was measured using a 3D optical surface roughness analyzer (Contour GT-X3 BASE, Brucker, Germany) at 9 locations per specimen. The average roughness (Ra) and maximum roughness (Rz) were calculated. The objective magnification was 20 ×, the zoom value was 2 ×, and the measurement field was 218 × 164 μm^2^.

### Scanning electron microscopy (SEM)

The surface topography was observed using a field emission scanning electron microscope (JEOL-7800F, JEOL, Japan) at an acceleration voltage of 15 kV. In the analysis, 1 specimen was randomly selected from each group and sputter-coated with gold–palladium for 180 s.

### Statistical analysis

All statistical analyses were performed using SPSS v25 (IBM SPSS Statistics, IBM, USA). Shapiro‒Wilk and Levene's tests were performed to verify the normality and homogeneity of variance, respectively. The FS, Vickers hardness, and surface roughness values were analyzed via one-way ANOVA, and Tukey’s post hoc test was performed to detect multiple comparisons among the groups. The significance level was set to α = 0.05. All of the methodologies were reviewed by an independent statistician.

## Results

### Biaxial flexural strength

The SMA group exhibited the highest FS value (1144.97 ± 168.1 MPa), followed by the SMC (944.58 ± 141.38 MPa), AMA (905.02 ± 111.38 MPa), and AMC groups (763.55 ± 68.69 MPa). In addition, the AM groups showed statistically lower FS values than those of the SM groups under the same surface treatments (p = 0.002 in the control group and p < 0.001 in the air-abrasion group), whereas the air-abrasion group showed statistically higher FS values than those of the control group with the same materials (p = 0.019 in the AM group and p < 0.001 in the SM group) (Table 2).

**Table 2:** Mean values ± standard deviations of biaxial flexural strength (MPa) and Vickers hardness (HV).

Group	Flexural strength (MPa)	Vickers hardness (HV)
	Mean ± SD	Mean ± SD
AMC	763.55 ± 68.69^a^	1259.61 ± 37.01^a^
AMA	905.02 ± 111.38^b^	1256.52 ± 43.88^a^
SMC	944.58 ± 141.38^b^	1273.03 ± 43.86^ab^
SMA	1144.97 ± 168.1^c^	1286.33 ± 25.61^b^

Different letters in columns indicate statistically significant differences (p < 0.05) among tested groups.

*AMC* additive manufacturing control group, *AMA* additive manufacturing air abrasion group, *SMC* subtractive manufacturing control group, *SMA* subtractive manufacturing air abrasion group.

### Weibull distribution

**Figure 1:**
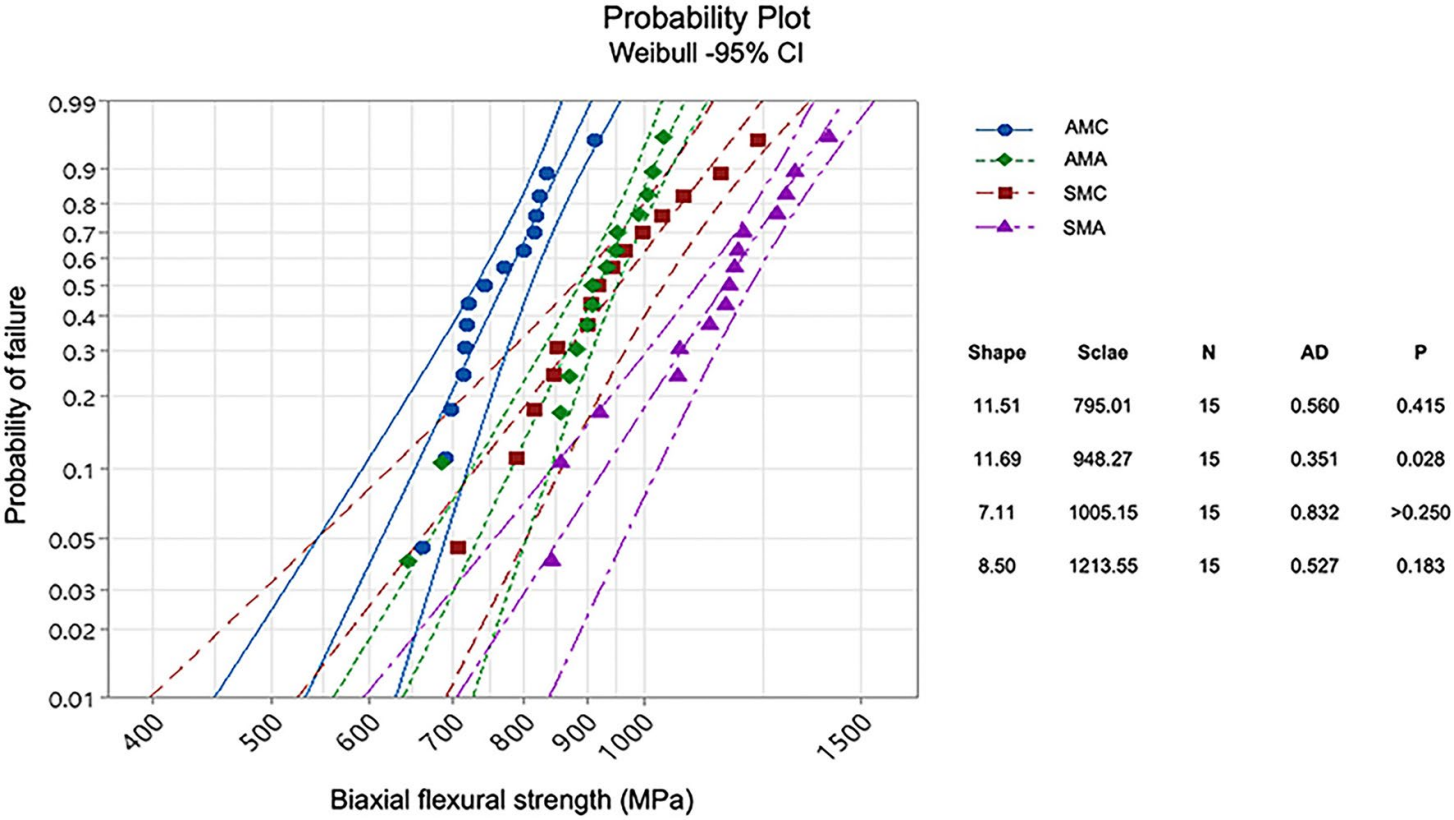
Weibull plot of biaxial flexural strength with 95% confidence bands.

The Weibull distribution showed that the SMA group had the highest scale value of 1213.55 MPa, with the highest shape value of 11.69 in the AMA group (Table 3). The graph of the SMA group on the far right indicates that it had the highest scale value, and the slope of the graph of the AMA group was the steepest, indicating that it exhibited the highest shape value (Fig. 1).

**Table 3:** Weibull parameters of all groups.

Group	Shape	95% CI at shape	Scale (B63.2)	95% CI at scale
AMC	11.51	7.97–16.63	795.01	758.73–833.01
AMA	11.69	7.69–17.77	948.27	906.37–992.11
SMC	7.11	4.90–10.31	1005.15	931.98–1084.08
SMA	8.50	5.69–12.71	1213.55	1139.88–1291.99

### Vickers hardness

In both the AM and SM groups, there was no significant difference in the Vickers hardness values between the control and air-abrasion groups. However, when compared in terms of the manufacturing method, the Vickers hardness of the AMA group (1256.52 ± 43.88 HV) was significantly lower than that of the SMA group (1286.33 ± 25.61 HV) (p = 0.002) (Table 2).

### X-ray diffraction (XRD)

**Figure 2:**
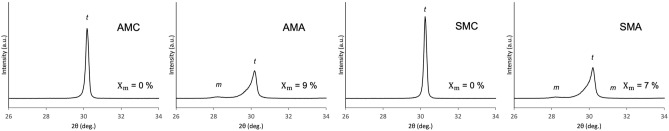
X-ray diffraction graphs of all groups. *m* monoclinic phase; *t* tetragonal phase; *X*_*m*_ monoclinic phase content.

The XRD patterns of the AMC and SMC groups showed only the tetragonal phase. In the AMA group, the monoclinic peak was observed at 2Ø = 28.2°, and the tetragonal peak was observed at 2Ø = 30.3°. In the SMA group, the monoclinic peak was observed at 2Ø = 28.2° and 2Ø = 31.4°, and the tetragonal peak was observed at 2Ø = 30.3° (Fig. 2). The monoclinic phase content (${\mathrm{X}}_{\mathrm{m}}$) was 9% in the AMA group, which was higher than that in the SMA group (7%).

### Surface roughness

**Figure 3:**
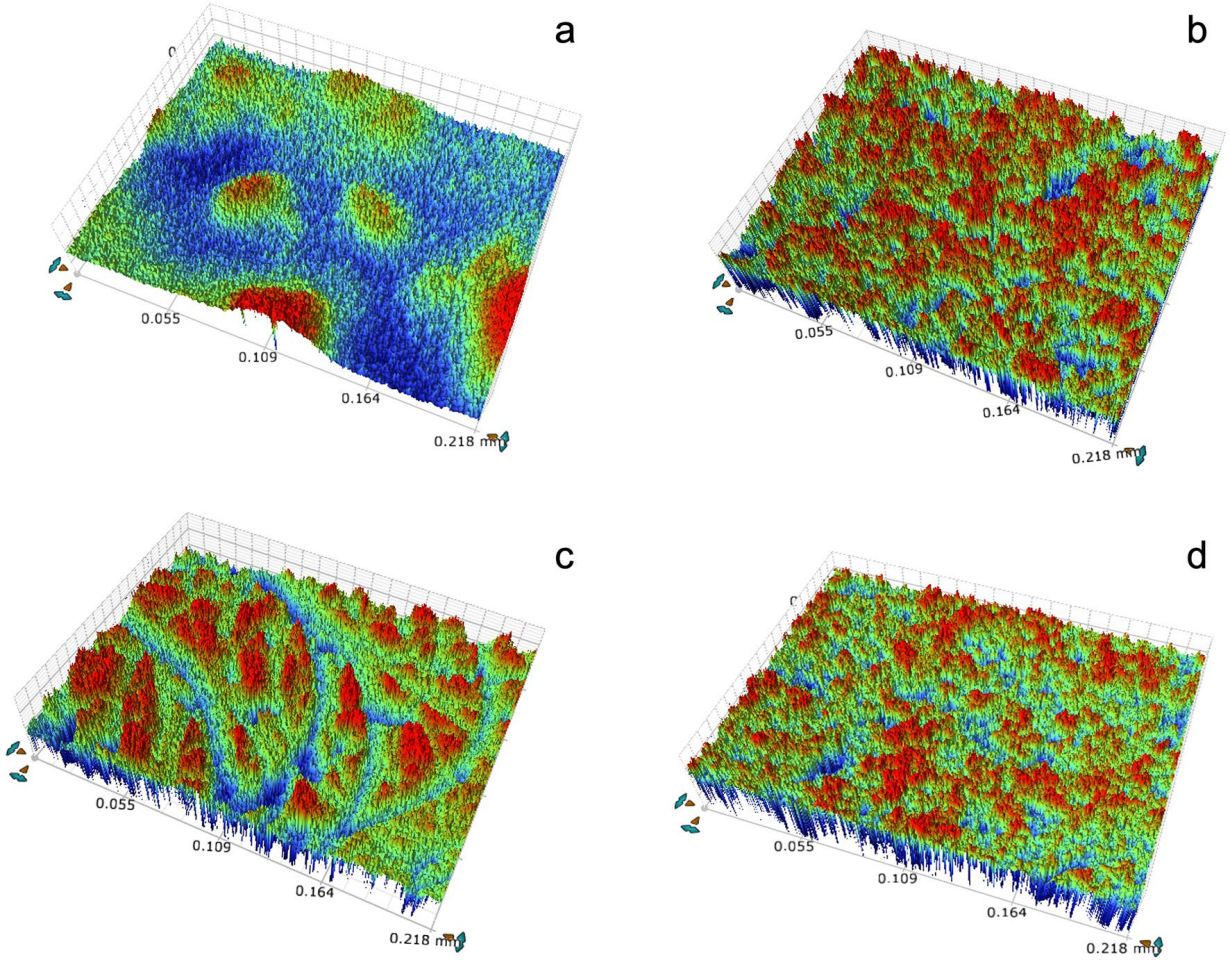
Surface roughness images. (**a**) AMC, (**b**) AMA, (**c**) SMC (**d**) SMA.

The surface roughness value, Ra, of the AMC group (0.40 ± 0.09 μm) was significantly lower than those of the other groups, which increased in the following order: SMA (0.50 ± 0.03 μm), AMA (0.55 ± 0.03 μm), and SMC (0.57 ± 0.08 μm) (p < 0.001). The Rz value of the AMC group (3.62 ± 0.63 μm) was significantly lower than that of the AMA group (9.37 ± 4.29 μm) (p < 0.001), but there was no significant difference between the Rz values of the SM groups. Although the Rz value of the AMC group (3.62 ± 0.63 μm) was significantly lower than that of the SMC group (5.79 ± 0.48 μm) (p = 0.024), the Rz value of the AMA group (9.37 ± 4.29 μm) was significantly higher than that of the SMA group (6.31 ± 0.94 μm) (p = 0.001) (Table 4, Fig. 3).

**Table 4:** Mean values ± standard deviations of surface roughness (Ra, Rz).

Group	Ra (μm)	Rz (μm)
	Mean ± SD	Mean ± SD
AMC	0.40 ± 0.09^a^	3.62 ± 0.63^a^
AMA	0.55 ± 0.03^c^	9.37 ± 4.29^c^
SMC	0.57 ± 0.08^c^	5.79 ± 0.48^b^
SMA	0.50 ± 0.03^b^	6.31 ± 0.94^b^

Different letters in columns indicate statistically significant differences (p < 0.05) among tested groups.

### Scanning electron microscopy (SEM)

**Figure 4:**
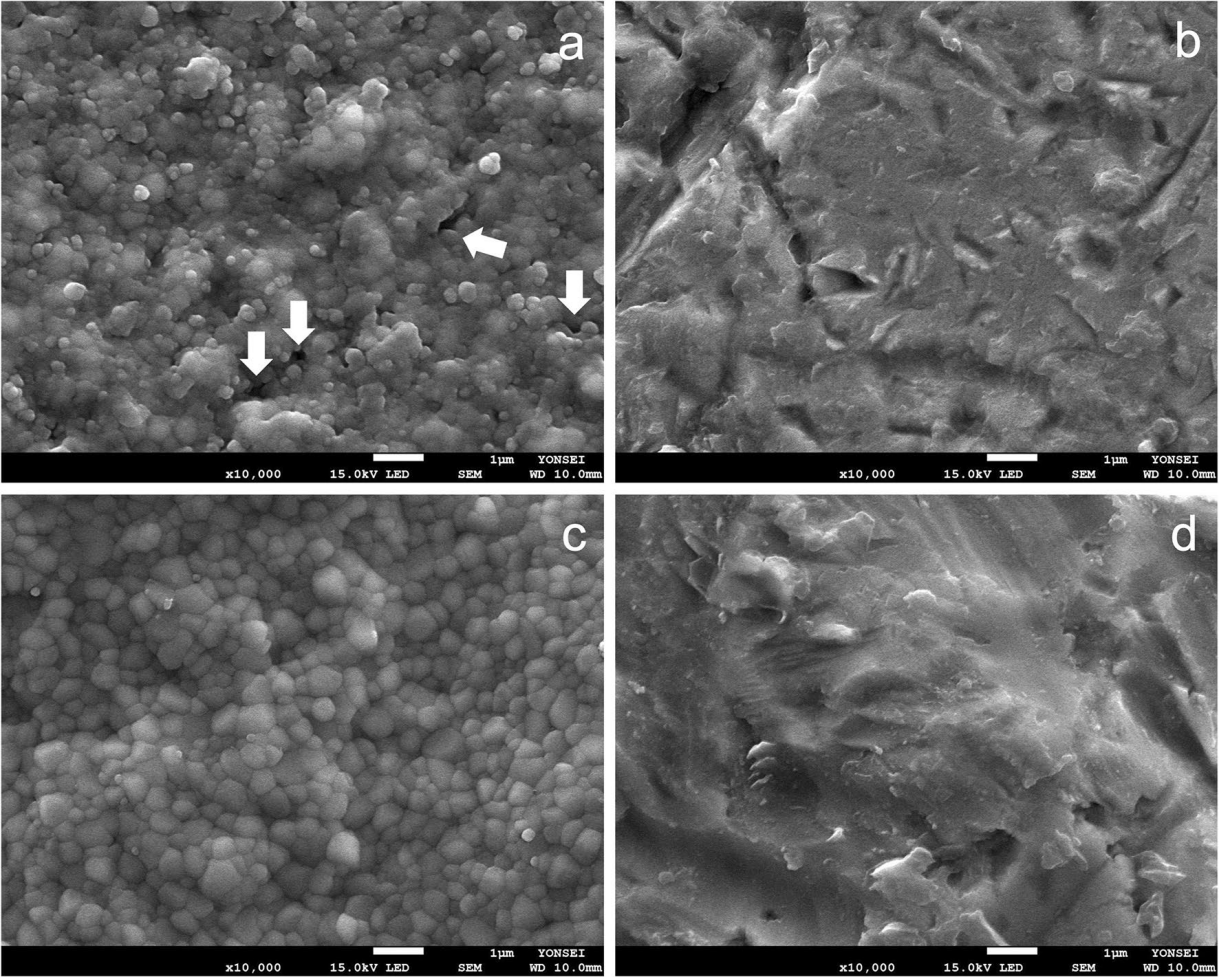
Scanning electron microscopy (SEM) photographs × 10,000. (**a**) AMC, (**b**) AMA, (**c**) SMC, (**d**) SMA. Pores are shown with white arrows.

The SEM analysis showed that the grain size of the SMC group was relatively uniform, while the AMC group comprised large and small grains, and many pores or grain pull-outs were observed. The AMA group showed an irregular surface topography with many scratches and deep grooves, whereas the SMA group exhibited a relatively smooth and irregular surface, but some deep pits were observed (Fig. 4).

## Discussion

This study compared the mechanical properties of zirconia fabricated via AM and SM technologies and evaluated whether air-abrasion surface treatment with Al_2_O_3_ affected the mechanical properties of both zirconia groups. The first null hypothesis was partially rejected, as significant differences in the FS were found among the AM and SM groups, but not all groups were significantly different in terms of their Vickers hardness. Additionally, the second null hypothesis was partially rejected because the air-abrasion surface treatment significantly affected the FS of zirconia fabricated by AM and SM technologies but not the Vickers hardness.

In the present study, the zirconia specimens fabricated by AM technology had significantly lower FS values than those of the specimens fabricated by SM technology. These results are similar to those of other previous studies reporting that the lower FS of AM groups may result from the weak areas of the boundaries between stacked layers, where residual stress is likely to cause cracks or delamination^12^. Another study reported that a milled zirconia group (914 ± 68.12 MPa), which was similar to that in this study, attained a significantly higher FS than the AM zirconia group (320.32 ± 40.55 MPa)^2^, which is much lower than the corresponding FS value in this report.

Moreover, a study by Zhai^12^ regarding the aging effects on Y-TZP printed by both SM and AM technologies reported that the FS values were significantly higher for the SM group (1273.3 ± 170.2 MPa) than for both the DLP (845.6 ± 183.5 MPa) and SLA group (776.7 ± 77.0 MPa). The FS value of AM zirconia was similar to that of this study, but the milled zirconia showed a higher FS. These differences in FS values might be explained by inconsistencies in the FS testing methods, zirconia manufacturing processes, polishing procedures, sintering shrinkage and chemical composition, and the raw material grain size.

According to ISO 6872, dental ceramics are specified to have an FS of at least 300 MPa for a single unit and 800 MPa for a four-unit prosthesis; in addition, previous studies have reported FS values of 3Y-TZP manufactured by subtractive techniques of approximately 800 to 1200 MPa^1,2,17,22^. The FS values of all groups in the present study were within this range.

Our study showed that the FS was significantly increased by approximately 18% for the AM group and 21% for the SM group when air abrasion of the zirconia surface was performed at 0.2 MPa with 50 μm alumina particles. This increase can be attributed to toughening transformation from the tetragonal to the monoclinic phases, which induced a residual compressive stress that prevented crack development, resulting in a strength increase^30^. In the control group, the m-phase was not observed, whereas in the air-abrasion group, the m-phase of the AMA group was 9% and that of the SMA group was 7% within the standard limit of ISO 13356 that the m-phase content of zirconia should be less than 20% before low-temperature degradation (LTD) and 25% after LTD. The FS of air-abraded zirconia is considered a result of the trade-off between the damage and residual compressive stress generated by particle impact^31^. When surface flaws induced by the air-abrasion procedure appeared to remain confined within the transformation layer, they were probably compensated for by the 4% grain volume increase during the phase transformation, creating a layer of residual compressive stresses^32^. Therefore, parameters related to air abrasion should be applied with caution to prevent surface weakness.

The Weibull statistics characterize the structural reliability of brittle dental materials^27,33^. Considering solely the mean flexural strength is insufficient for accurately characterizing ceramic properties, and the significant variability in the failure strength due to flaws introduced during specimen processing should be considered^34,35^. Thus, Weibull analysis related to the flaw-size distribution was employed to consider the strength variability^34,36^. Regarding materials with low Weibull shape values, fractures can occur within a large portion of the specimens, while fracture origins cluster in areas of the highest stress with high Weibull shape values^27^. The shape value for most dental ceramics is reported to vary between 5 and 15, and a lower Weibull shape indicates greater variability and less reliability in the strength due to flaws and defects in the material^33^. The 3D printing procedure, cleaning, debinding, sintering process, and porosity content can affect the Weibull shape^16,20^. In addition, aging or LTD inducing compressive stress zones through t-m transformation at the surface can increase the Weibull shape and mechanical properties^2,37^. In this study, the Weibull shapes were 11.51 ~ 11.69 for the AM group and 7.11 ~ 8.50 for the SM group. Generally, the Weibull shape of SM is higher than that of AM^2,4,16,17^, which is explained by the imperfection of the internal material of AM zirconia^16^. However, a few previous studies have shown controversial results^5^. These discrepancies related to the shape of Weibull can be caused by inconsistencies in the testing methods, including the printing angle, grain size, and polishing method. The Weibull scale indicates the strength value at a probability of failure of 63.2%^27^; therefore, the change in the Weibull scale can be interpreted in terms of the material strength. The shape and scale values of the air-abrasion groups were higher than those of the control groups in this study, which may indicate that the air-abrasion surface treatment forms compressive zones by increasing the m-phase, resulting in the improvement in the mechanical properties of zirconia without causing unstable flaws or substrate damage^23,31^.

The Vickers hardness indicates a material's ability to withstand plastic deformation, material deterioration, and fatigue. It is related to the material survival rate and generally depends on the porosity and grain size^38–40^. The Vickers hardness of the AMC group tended to be lower than that of the SMC group with no significance, but that of the AMA group was significantly lower than that of the SMA group in this study; hence, it can be inferred that the manufacturing method affected the Vickers hardness. A previous study suggested that the presence of large pores on the surface of tested DLP zirconia was a reason why the Vickers hardness of AM zirconia was 5% lower than that of milled zirconia^38^, which agrees with our findings that there were many irregular pores in the SEM images of the AMC group. In contrast, in the air-abrasion groups, where no surface pores were observed, it was determined that the intrinsic strength of the material affects the Vickers hardness, implying that additional research is needed. The Vickers hardness of the AM group found in this study was in the range of 1248–1261 HV, which is in agreement with the hardness of zirconia manufactured by SM reported in previous studies^6,38^. In this study, there were no significant differences between the AMC and AMA groups or between the SMC and SMA groups in terms of the Vickers hardness, indicating that air abrasion did not significantly affect this property. However, a previous study reported that the Vickers hardness of SM zirconia was significantly increased from 1219.83 ± 94.11 to 1940.63 ± 458.38 HV by air abrasion with a 50 μm particle size under 4-bar pressure for 15 s from a distance of approximately 10 mm^41^. The differences in the results may be related to the test parameters, such as the air pressure and time. However, 4-bar (0.4 MPa) pressure was reported as the value at which air abrasion decreased the FS^22^.

The surface roughness appeared to increase significantly by air abrasion in the AM group. However, the SM group showed an increase in Rz but a decrease in Ra, which means that the air-abrasion treatment decreased the average surface roughness but increased the maximum roughness of SM zirconia due to some greater pits. Further studies are needed to obtain an appropriate air-abrasion protocol for AM zirconia under strict control in terms of sample sizes and test techniques, as well as to verify various related factors, such as the zirconia particle size and porosity. The limitation of the current study is that the effects of low-temperature degradation or aging of zirconia were not considered during the experimental evaluation of zirconia fabricated via AM technology.

## Conclusion

Within the limitations of this study, the following conclusions can be drawn: the FS and Vickers hardness of zirconia fabricated by AM technology were lower than those of zirconia fabricated by SM technology but were within the clinically acceptable ranges. Air-abrasion surface treatment increased the monoclinic phase content, FS, and surface roughness of the AM group.

## Data Availability

The authors declare that the data are available.

## Abbreviations

3Y-TZP: 3% Yttria-tetragonal zirconia polycrystals
CAM: Computer-aided manufacturing
SM: Subtractive manufacturing
CNC: Computer numerical control
AM: Additive manufacturing
SLA: Stereolithography apparatus
DLP: Digital light processing
FS: Flexural strength
10-MDP: 10-Methacryloyloxydecyl dihydrogen phosphate
XRD: X-ray diffraction
SEM: Scanning electron microscopy
